# Relation of pandemics with solar cycles through ozone, cloud seeds, and vitamin D

**DOI:** 10.1007/s11356-022-22982-1

**Published:** 2022-09-23

**Authors:** Kwan Chul Lee, Jung Sun Kim, Young Sil Kwak

**Affiliations:** 1 Korea Institute of Fusion Energy, 169-148 Gwahak-ro, Yuseong-gu, Daejeon, 34133 Korea; 2 Konyang University Medical Campus, 158 Gwanjeodong-ro, Seo-gu, Daejeon, 35365 Korea; 3 Korea Astronomy and Space Science Institute, 776 Daedeok-daero, Yuseong-gu, Daejeon, 34055 Korea

**Keywords:** Pandemic, Vitamin D, Solar activity, Ozone, Cloud seed

## Abstract

The global records of infectious diseases, including Western and Eastern documents from 1825 to 2020, during which sunspot observations are considered reliable, show that 27 of the 34 pandemic outbreaks were coincident with sunspot number maxima or minima. There is evidence that the intensity of galactic cosmic rays is anti-correlated with solar activity and that cloud seed formation is accelerated by galactic cosmic rays. There are a substantial number of research papers showing the relationship between COVID-19 and vitamin D deficiency. The data analysis of ozone thickness measured based on NASA satellite observations revealed that ozone thickness has 11-year and 28-month cycles. Because the 11-year cycles of ozone thickness and cloud seed attenuation are anti-correlated, when either one becomes extremely thick, such as at the maximum or minimum point of solar activity, UV radiation is over-attenuated, and human vitamin D deficiency is globally increased. This finding explains the coincidence of pandemic outbreaks with the extrema of the sunspot numbers. Vitamin D supplementation can be an effective countermeasure against the spread of infectious diseases, which is a paramount importance to global society. Future pandemic forecasting should include the 11-year and 28-month cycles of UV radiation. This founding completes the relationship between solar activity and human health through the earth’s environment.

## Introduction

The core of the sun comprises high-density and high-temperature plasma; hence, nuclear fusion reactions generate solar energy, and the magneto-hydrodynamics (MHD) of the plasma induces periodic activity known as the solar cycle. The understanding of the solar activity that induces solar storms and aurora by the plasma interaction with the earth’s atmosphere is improved (Lee 2017). There have been studies on the relationship between the solar cycle and pandemics since the first proposal by Hope-Simpson (Hope-Simpson 1978; Hoyle and Wickramasinghe 1990). While many other mechanisms were discussed for the connection between the solar cycle and pandemics, such as mutations induced by radiation, vitamin D is introduced as an important element in this connection (Cannell et al. 2008). Hayes proposed a hypothesis that ozone circulation controls earth ultraviolet (UV) exposure indirectly so that the solar cycle is related to vitamin D levels in humans (Hayes 2010). However, this hypothesis was incomplete to explain pandemic occurrences coincident with the solar cycle both at maximum and minimum (Qu 2016) since it did not include the cloud seed generation by galactic cosmic rays, which were discovered later (Kirkby et al. 2016). Infectious diseases such as COVID-19 can spread with manifold transmission dynamics such as temperature or humidity and environmental conditions. A study found a relationship of population density and import–export with COVID-19 infections (Bontempi and Coccia 2021). There are other elements than ozone and cloud seed between the sun and human skin as UV radiation propagates, such as air pollution. The higher air pollution resulted in higher COVID-19 infections (Srivastava 2021; Coccia 2021). And the seasonal dependence of COVID-19 infection (low in summer and high in fall/winter) is confirmed (Coccia 2022a). These two influences on COVID-19 are in accord with the mechanism that low UV exposure and vitamin D deficiency increase infections. This narrative review is focused on the underlying mechanisms of the 11-year solar cycle modulating the damage of the infectious disease.

## Study design

First, a statistical reassessment of the association between sunspot activity and the timing of the infectious disease outbreaks is performed. This is important since the previous assessment was carried out with a small number of samples from the western world only. Second, the studies on vitamin D protection against respiratory tract infections are reviewed since this relation is intensively investigated independently from the solar activity, especially on COVID-19. Third, the ozone thickness observation data is analyzed to estimate the role of ozone variation induced by solar activity on the pandemics. Lastly, the influence of cloud seed generation by galactic cosmic rays on the UV attenuation is discussed because the role of cloud seed on the UV attenuation can complete the mechanism that relates the solar activity with the pandemic occurrence at the maximum and minimum of sunspot number.

### Sample and data

The samples of influenza that occurred in Europe were taken from 10 records that were summarized in a recent statistical work (Towers 2017), and the samples of the epidemic that occurred in Korea were taken from the Veritable Records of the Joseon Dynasty and a modern record. The samples from the Veritable Records of the Joseon Dynasty were summarized in a recent report (Lee 2019). The data for the ozone thickness variation was taken from the measurement by the microwave limb sounder (MSL) on NASA’s AURA satellite (Ziemke et al. 2006).

### Data analysis procedure

The data for the ozone thickness were taken for latitude of 8° north (averaged between 7 and 9°) and 30° north (averaged between 29 and 31°), the latitude of 8° corresponds Ebola outbreak location, and 30° corresponds COVID-19 outbreak location. And the data from Mar. 1 to Mar. 10 were taken and averaged to represent as the value of each year. The data are available from the year 2005, and March 10 2020 is the latest date when the data were taken. And the effect of sun’s incidence angle is included for the UV attenuation in Figures 2 and 3.

## Results and discussion

### Association between the solar cycle and pandemic occurrence

**Fig. 1:**
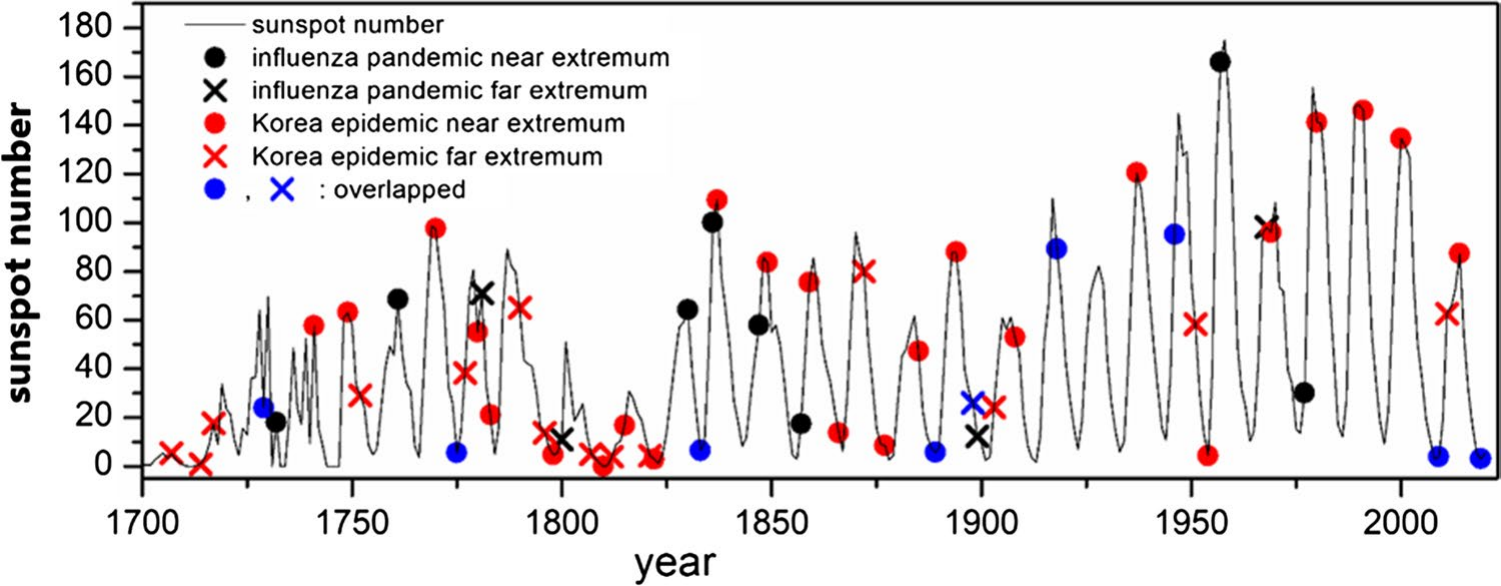
Comparison of recorded pandemic year and sunspot number (SSN) data. Sunspot number data from 1700 to approximately 1995 are group SSN (Hoyt and Schatten 1998). SSN data from 1996 to approximately 2020 are R sunspot numbers adjusted to match the group SSN (NASA 2020). Black points are from Towers’ data (Towers 2017) plus COVID-19. The red points are from the Veritable Records of Joseon Dynasty (1700–1910) (Lee 2019) and a modern report for 1910–2020 (Chung 2016; Schofield 1919). Blue points are overlapping data for both Western and Korean records

When a study on the connection between solar activity and the influenza outbreak suggested that the pandemics take place on the extremum or ± 1 year of sunspot number (Qu 2016), an evaluation study with strict statistical criteria on this claim concluded that there was no correlation (Towers 2017). Towers' conclusion has important implications. First, when dealing with the correlation between two events, data distortion can occur. After correcting for these distortions, the correlation between the solar cycle and the pandemic was not clear. For the causal relationship, it is obvious that the outbreak of infectious diseases does not affect the solar cycle, and not all pandemics are related to the solar cycle. For example, in America as a new world, pandemics have occurred due to transmission by people from the old world. Therefore, it is difficult to explain all outbreaks of infectious diseases by a hypothesis such as the mutations induced by the neutron from cosmic rays (Bell 2022). Instead, when an epidemic occurs independently of the solar cycle, it is possible that the damage of the epidemic could be greater if the timing is close to the extremum of the sunspot cycle. Towers dealt with influenza pandemics from 1700 to 2014, with a total of 20 occurrences, 15 of which were between − 1 and + 1 year from the maximum or minimum points of the sunspot number. If a pandemic occurs independently of the solar cycle, there have been 57 extrema in 315 years; thus, the probability of occurring between ± 1 of extrema is 170/315. According to the binary event probability, the probability of 15 of 20 occurrences was 3.3%. This can be converted to an odds ratio (O.D.) of 15 × 140/155 × 5 = 2.7. In Towers’ study, 20 occurrences were carefully selected from ten records, but this is still a small number to judge the relationship. If the case of COVID-19 is added to the Towers’ data, 16 out of 21 cases are true, and the probability of this happening by chance drops to 2.4%. On the other hand, the association between sunspots and infectious diseases is not limited to influenza, and the incidence of other infectious diseases, such as smallpox and plague, has also been suggested to be associated with the solar cycle (Wickramasinghe et al. 2017). The Towers’ study covered records from Europe, but epidemics have also occurred elsewhere on the planet, and there are records of them. The “Veritable Records of Joseon Dynasty,” which records events from 1413 to the beginning of the twentieth century in Korea, is the longest record of a single dynasty. Considering the outbreaks of infectious diseases appearing in this document, 45 epidemics were recorded from 1700 to 1910 (Lee 2019). Including epidemics that occurred after 1910 through modern records (Chung 2016; Schofield 1919) and excluding eight cases overlapping with Towers’ data (global pandemics such as those in 1918 and 2019 should be counted as one), 39 cases can be added to Towers’ data. As shown in Fig. 1, there were 40 cases in which the number of sunspots was at its extreme value within ± 1 year out of 59 occurrences. The details of these 59 cases are listed in Table 1. The probability that these 40 out of 59 cases occurred by chance is 1.2%. The probability of the data used by Towers has decreased by nearly one-third. As the data size increased, the association between the two phenomena also increased. Judging the relationship between epidemic outbreaks and the number of sunspots can improve as more data becomes available.

**Table 1:** List of epidemics that occurred from 1700 to approximately 2020

No	Year	Nearest extremum	Ref	Disease	Figure 1 symbol	No	Year	Nearest extremum	Ref	Disease	Figure 1 symbol
1	1707	1705 (max)	[1]	N.A	✕	31	1849	1848 (max)	[1]	N.A	○
2	1714	1712 (min)	[1]	N.A	✕	32	1857	1856 (min)	[1]	Influenza	○
3	1717	1719 (max)	[1]	N.A	✕	33	1859	1860 (max)	[21]	N.A	**○**
4	1729	1730 (max)	[1]/[2]	Influenza/N.A	**○**	34	1866	1867 (min)	[1]	N.A	○
5	1732	1733 (min)	[2]	Influenza	**○**	35	1872	1870 (max)	[1]	N.A	✕
6	1741	1741 (max)	[1]	N.A	**○**	36	1877	1878 (min)	[1]	N.A	**○**
7	1749	1749 (max)	[1]	N.A	**○**	37	1885	1884 (max)	[1]	N.A	○
8	1752	1749 (max) 1755 (min)	[1]	N.A	✕	38	1889	1889 (min)	[1]/[2]	Influenza/N.A	**○**
9	1761	1761 (max)	[2]	Influenza	**○**	39	1894	1894 (max)	[1]	N.A	○
10	1770	1769 (max)	[1]	N.A	**○**	40	1898	1901 (min)	[1]/[2]	Influenza/N.A	✕
11	1775	1775 (min)	[1]/[2]	Influenza/N.A	**○**	41	1899	1901 (min)	[2]	Influenza	✕
12	1777	1779 (max)	[1]	N.A	✕	42	1903	1901 (min)	[1]	N.A	✕
13	1780	1779 (max)	[1]	N.A	**○**	43	1908	1907 (max)	[1]	N.A	**○**
14	1781	1779 (max)	[2]	Influenza	✕	44	1918	1917 (max)	[2]/[3]	Influenza/Influenza	**○**
15	1783	1784 (min)	[1]	N.A	**○**	45	1937	1937 (max)	[4]	Tuberculosis	**○**

16	1790	1787 (max)	[1]	N.A	✕	46	1946	1947 (max)	[2] /[4]	Influenza/N.A	**○**
17	1796	1798 (min)	[1]	Influenza	✕	47	1951	1954 (min)	[4]	Smallpox	✕
18	1798	1798 (min)	[1]	N.A	**○**	48	1954	1954 (min)	[4]	Tuberculosis	**○**
19	1800	1798 (min)	[1]/[2]	Influenza/N.A	✕	49	1957	1958 (max)	[2]	Influenza	**○**
20	1807	1810 (min)	[1]	N.A	✕	50	1968	1970 (max)	[2]	Influenza	✕
21	1810	1810 (min)	[1]	N.A	**○**	51	1969	1970 (max)	[4]	Cholera	**○**
22	1812	1810 (min)	[1]	N.A	✕	52	1977	1976 (min)	[2]	Influenza	**○**
23	1815	1816 (max)	[1]	N.A	**○**	53	1980	1979 (max)	[4]	Cholera	**○**
24	1821	1823 (min)	[1]	N.A	✕	54	1991	1990 (max)	[4]	Cholera	**○**
25	1822	1823 (min)	[1]	N.A	○	55	2000	2000 (max)	[4]	Measles	**○**
26	1830	1830 (max)	[2]	Influenza	○	56	2009	2008 (min)	[2]	Influenza	**○**
27	1833	1833 (min)	[1]/[2]	Influenza/N.A	○	57	2011	2008 (min)	[4]	Measles	✕
28	1836	1837 (max)	[2]	Influenza	**○**	58	2014	2014 (max)	[4]	Measles	**○**
29	1837	1837 (max)	[1]	N.A	**○**	59	2019	2019 (min)	[5]	COVID-19	**○**
30	1847	1848 (max)	[2]	Influenza	**○**						

[1]: Lee (2019); [2]: Towers (2017); [3]: Schofield (1919); [4]: Chung (2016); [5]: Borsche et al. (2021)

A comparison of the sunspot cycles and the years in which past epidemic outbreaks occurred is summarized in Fig. 1. It shows that the reliability of the sunspot cycle records before 1825 is low, which was confirmed by a sunspot cycle analysis (Clette et al. 2015). This is because the sunspot number observation method was not well regulated before 1825, and the solar activity was weakened by the Maunder minimum and Dalton minimum. Considering only data since 1825, there have been a total of 34 epidemic outbreaks, with 27 occurring near the extrema of sunspot numbers. The probability of this occurrence by chance is only 0.16%. This can be converted to an O.D. of (27 × 82)/(81 × 7) = 3.9. Even if the solar activity does not directly cause an epidemic, it is possible that the damage is amplified by the underlying mechanism of solar activity. A study on epidemics that occurred between 1750 and 2020, most of which are not included in Fig. 1, also showed a strong correlation with solar cycle extrema (Nasirpour et al. 2021). Another important phenomenon is that, among the 40 events that occurred near the extrema between 1700 and 2021, 24 occurred near the maxima and 16 occurred near the minima. There must be a mechanism linked to these epidemics, both when the sun’s activity is strong and when it is weak.

### Relationship between vitamin D deficiency and acute respiratory disease

Studies showing that vitamin D plays an important role in the immune system against infections date back to at least the 1990s (Lemire 1992). Since then, studies have attempted to determine why many respiratory infections occur in winter when the incidence of ultraviolet rays is weak and vitamin D deficiency is high (Cannell et al. 2006). On the one hand, studies have shown that vitamin D is an important factor; on the other hand, a study in New Zealand showed that supplementation with vitamin D has no effect on respiratory disease infection (Murdoch et al. 2012). Since this was a 2-year clinical study of 322 patients, it is necessary to examine why this study yielded a negative result. There are two main reasons for this finding. First, even in the winter of the Southern Hemisphere, due to the high UV incidence in New Zealand, where the clinical trial was conducted, the blood vitamin D concentration does not fall below an average of 30 ng/mL. The average blood vitamin D concentration of the test subjects before the clinical trial was 29 ng/mL. A later clinical study conducted in the UK with a total of 11,000 patients in 25 cases revealed that vitamin D supplementation was effective mainly when the blood vitamin D concentration was lower than 10 ng/mL (Martineau et al. 2017). Therefore, the vitamin D concentration in the New Zealand study was already too high to be affected by vitamin D supplementation. The second reason is that the method of supplementing vitamin D was to take 100,000–200,000 IU once a month in the New Zealand study, but because vitamin D has limited absorption and needs to be activated in the liver and kidney over a period of time, taking a small amount daily is the most effective, and taking a large amount intermittently is not effective. This fact was also supported by the UK study (Martineau et al. 2017). After the outbreak of COVID-19, many studies have shown that vitamin D deficiency increases the likelihood of COVID-19 infection, and vitamin D deficiency increases the chances of going to the intensive care unit (ICU). The probability of infection with COVID-19 was found to be inversely proportional to the vitamin D blood concentration by a study of more than 190,000 American patients (Kaufman et al. 2020). The mortality risk of patients with vitamin D deficiency is 7 times higher than that of patients with normal vitamin D levels (Jain et al. 2020). Another research showed that patients with less than 20 ng/mL are 14 times more likely to have the severe or critical disease than patients with 40 ng/mL or higher (Dror et al. 2022). To date, more than 1090 studies on the relationship between vitamin D deficiency and COVID-19 have been published, including reviews showing a positive correlation (Borsche et al. 2021; Mercola et al. 2020). A study showed that patients with darker skin had a 2.5 times higher COVID-19 infection rate, which is related to vitamin D deficiency (Meltzer et al. 2020). A previous study has shown that the number of COVID-19 deaths decreases with increasing UV radiation (Moozhipurath et al. 2020). Treatment with vitamin D as an active form in COVID-19 patients reduced the rate of intensive care unit admission by 25-fold (Entrenas Castillo et al. 2020). It can be concluded that although further studies are needed for the detailed mechanism, vitamin D plays a role in modulating innate immune function and protecting severe symptoms of COVID-19 (Latifi-Pupovci et al. 2022).

### Relationship between the solar cycle and the blocking of ultraviolet rays by ozone and cloud seeds

**Fig. 2:**
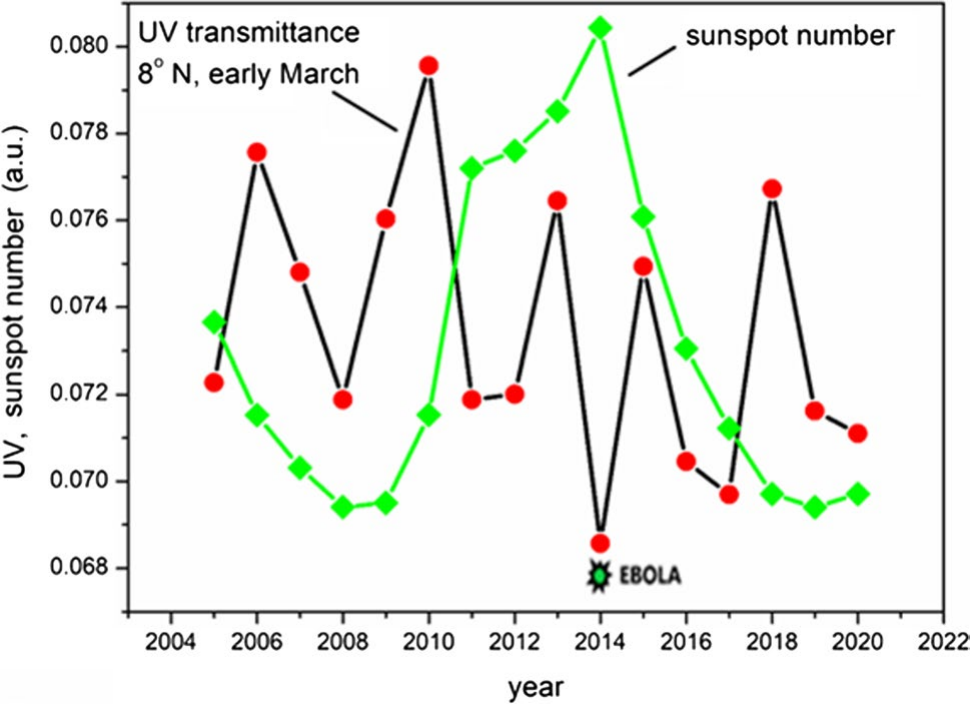
Transmittance variation of UV-B based on the ozone concentration measured by satellite detection for 8° north latitude (averaged between 7 and 9°) over 10 days of early March (averaged over March 1–10), and sunspot number variation

**Fig. 3:**
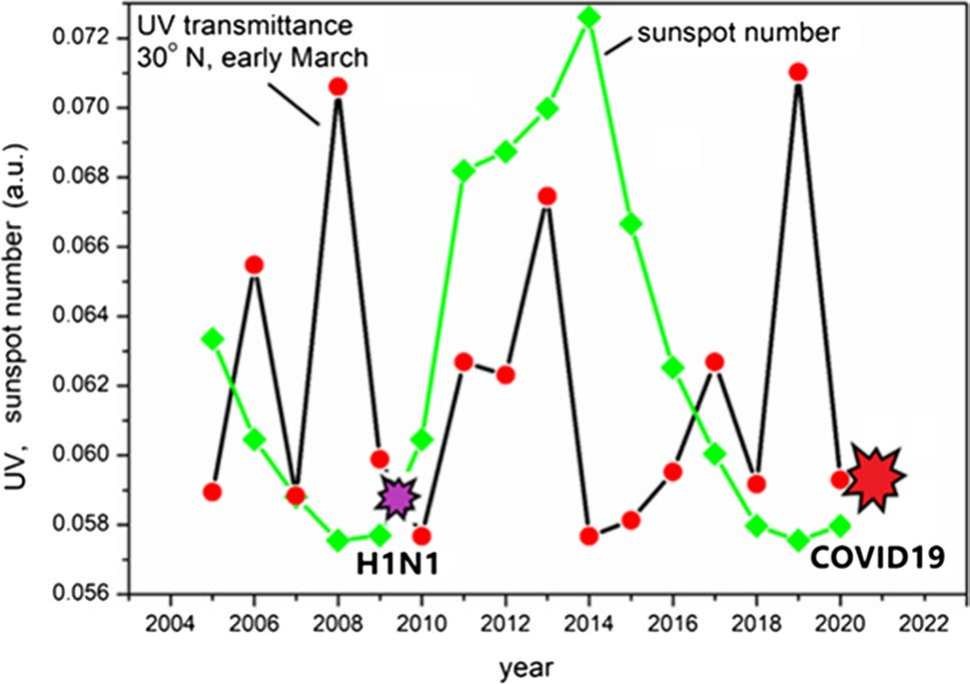
Transmittance variation of UV-B based on the ozone concentration measured by satellite detection for 30° north latitude (averaged between 29 and 31°) over 10 days of early March (averaged over March 1–10), and sunspot number variation

Ultraviolet B (wavelength of 280–315 nm), which produces vitamin D, is incident on Earth from the sun and is first absorbed by the ozone layer in the stratosphere. The rate of change in UV-B incidence from the sun according to the solar cycle is less than 1%. However, the ozone layer that blocks most of it is also generated by UV rays, so the amount of change in UV rays transmitted through the ozone layer is inversely proportional to the amount of change from the sun, and the rate of change is increased (up to 20%, as shown in Fig. 3). This anti-correlation between solar activity and UV-B transmittance through ozone was suggested in 2002 (Rozema et al. 2002). Therefore, UV-B light that passed through ozone at the high point of the solar cycle had the lowest value. Applying this logic can explain why an epidemic occurs at the highest point of the sunspot cycle. UV-B, which has passed through the ozone layer, is blocked again by particles in the lower part of the atmosphere, including aerosols and clouds. Cloud formation is determined by the flow of the Earth’s atmosphere and the resulting weather, so it is easy to think that it is not related to the solar cycle. However, it was recently discovered that galactic cosmic rays, consisting of charged particles from space, are involved in the formation of cloud seeds. When the organic vapor generated by Earth’s plants grows as a cloud seed, it grows 10–100 times faster if it is charged by interaction with galactic cosmic rays than when it is neutral (Kirkby et al. 2016). Another important fact is that galactic cosmic rays and solar wind have an anticorrelated relationship. When the solar wind is strong in the solar cycle, the space around Earth is under the influence of solar wind composed of relatively low-energy charged particles, and galactic cosmic rays composed of high-energy charged particles are scattered by the solar wind, decreasing the incident amount. Here, the low-energy charged particles are captured by Earth’s magnetic field. This phenomenon was discovered through observation (Richardson 2004). To date, the investigation of the variation in the amount of cloud coverage due to the solar cycle has not yielded a final explanation, with observations showing only a 2–5% variation (Pierce 2017). Although the variation in ion production caused by galactic cosmic rays during the solar cycle can be as large as 40%, the acceleration of cloud seed development causes smaller variations in cloud formation (Svensmark et al. 2016). There is a complex process that includes an opposite mechanism in which too many cloud seeds may reduce cloud formation. However, the particle number of cloud seeds ranging from 1 to 100 nm in size can vary according to the ionization rate. A change in the number of these particles can alter the attenuation only for light with a narrow wavelength range. Light with wavelengths that are too short is already scattered by air molecules, whereas light with wavelengths that are too long is not effective for Rayleigh scattering. This can be explained by the cross-section of Rayleigh scattering, which is inversely proportional to the 4th power of the wavelength and proportional to the 6th power of the particle size; a particle size greater than 10% of the wavelength is not suitable for Rayleigh scattering. If there is a substantial change in the number of particles of 1–100 nm, the attenuation of the 300 nm UV rays will be largely affected by it. The UV attenuation by aerosols is estimated to be as large as that by air (Deng et al. 2012). One of the reasons that the UV attenuation by the cloud seed is not recognized so far is that both UV rays and cloud seed are invisible to human eyes. A detailed investigation of the aerosol and cloud seed number change caused by the solar cycle has yet to be conducted. However, a recent simulation program that did not include this effect showed a deviation from the ground UV measurement when the solar activity was low in 2009 (Figure 8 of Kazadzis et al. 2016). This hypothesis can explain why vitamin D deficiency is increased at the low point of the solar cycle, which induces a more severe epidemic outbreak, because the cloud seed variation by solar cycle is larger than the cloud variation. The ozone layer and cloud seed are generally in balance, keeping the amount of ultraviolet rays constant by thinning one when the other becomes thicker. However, in the vicinity of the extrema, when one becomes extremely thick and the other is no longer thinner, the balance can be broken. Therefore, an outbreak of infectious diseases occurred at both the high and low points of the number of sunspots, as shown in Fig. 1. This mechanism explains why all the sunspot minimums such as Sporor minimum, Mounder minimum, and Dalton minimum in the middle age, coincided with pandemics (Wickramasinghe et al. 2017). Especially Wolf minimum was well matched with the most fatal pandemic recorded in history; the black death in the fourteenth century. The year 2020 was among the lowest points in terms of the number of sunspots over the last 100 years. Korea had its longest rainy summer in 2020 since observation began, and January 2020 in Daegu, Korea, where the number of COVID-19 deaths was high, was the second cloudy January in 110 years, with the first cloudy January occurring in 1919 when a deadly influenza probably related to the Spanish flu resulted in 140,000 casualties (Korea Meteorological Administration 2020; Schofield 1919). More cloud seeds induce more flooding when moisture is supplied. In the summer of 2020 in China, 60 million people were displaced by the largest flood in 22 years (Global Times 2020). In Belgium, October 2020, when the number of COVID-19 infections was the highest, was the second darkest October since the observation began (Amies 2020). Heavy rainfalls occurred in 2021 in Europe and Canada, regarded as the worst flood in 100 years (Morris et al. 2021), and once in a 500-year event (Winter 2021). The link between cloudiness in Lima, Peru, and the highest death toll from COVID-19 per million people is a subject to be researched. To determine whether the ozone layer thickness was related to the sunspot period, data from satellites taken since 2005 were summarized. The UV transmissions in Figs. 2 and 3 were obtained from the ozone thickness measurements and the effect of the change in thickness caused by the incidence angle of the sun was included. Similar to clouds, the ozone layer changes rapidly with regional variation and exhibits seasonal characteristics. Figure 2 shows the average value of UV transmission by ozone in early March every year at approximately 8°N. As shown in Fig. 2, the amount of ultraviolet rays was lowest at the peak of the sunspot period in 2014. That year, an Ebola outbreak occurred near 8° north latitude in West Africa (World Health Organization 2019).

Figure 3, which presents data measured at a latitude of 30°, reveals two important features. First, the amount of ultraviolet rays is not only lowered during the sunspot high (2014) but also relatively low near the sunspot lows. Second, a remarkable 2-year periodicity appears. The 2-year periodicity, known as QBO (quasi-biannual oscillation), exists in the convection of the stratosphere in which the ozone layer exists; thus, the ozone layer can be interpreted as being affected by the QBO (Baldwin et al. 2001). The results indicated in Figs. 2 and 3 agree with a study of ozone thickness relation with the solar cycle, in which an 11-year cycle is found only at the low latitude due to Brewer-Dobson circulation (Grytsai et al. 2020). And the unclear relation between ozone thickness and solar activity in middle and high latitudes is also found by the ground UV-B exposure study using a proxy of pollen grains (Jardine et al. 2020). Some results of the UV-B ground exposure study for the past showed a positive relationship with the solar cycle, which is opposite to the suggested ozone thickness response (Nevalainen et al. 2018). This can be explained by the cloud seed attenuation because the ground UV-B is attenuated by both the ozone and the cloud seed, and the ozone thickness relation with the solar cycle is unclear in the high latitude. The fact that the ozone layer is not thinning at the low point of solar activity means that vitamin D deficiency may occur more severely at the low point of the sunspot cycle, where UV rays are additionally blocked by the cloud seed. H1N1 flu and COVID-19 occurred at these low points (da Costa et al. 2020). Evidence of global reduction in UV intensity during the COVID-19 period appeared in the comparison of 12-month previously measured vitamin D blood levels and the COVID-19 infection rate (Kaufman et al. 2020). If the UV intensity was reduced by the extremely low solar activity as the minimum of cycle 25 approached in early 2020, there must have been a further decrease in the vitamin D level just before the COVID-19 infection from the previously measured level. This reduction magnitude should be different for patients living in different latitudes. The higher latitudes have weaker UV light and narrower UV time windows at midday. Therefore, the exposure time must be longer for people living in higher latitudes to have the same vitamin D level. The ratio of the required exposure time to the UV time window is higher for higher latitudes. When the reduction rates of UV intensity for high and low latitudes are the same, the probability of the extra reduction in exposure caused by the UV time window ending before the required exposure time, at an outdoor lunch, for example, is higher for higher latitudes. The data showed that the patient living at higher latitudes had a higher infection rate even though the previously measured vitamin D level was the same. The same tendency was found for the darker skin subgroup. This effect can be amplified if the exposure time is longer for the case of weaker UV intensity compared to other cases with the same multiplication value of UV intensity (W/m^2^) and exposure time. An experiment using LED UV light showed that the exposure time for lower intensity to achieve the same amount of vitamin D formation is practically longer than that in the higher-intensity case (Kalajian et al. 2017).

## Conclusions

**Fig. 4:**
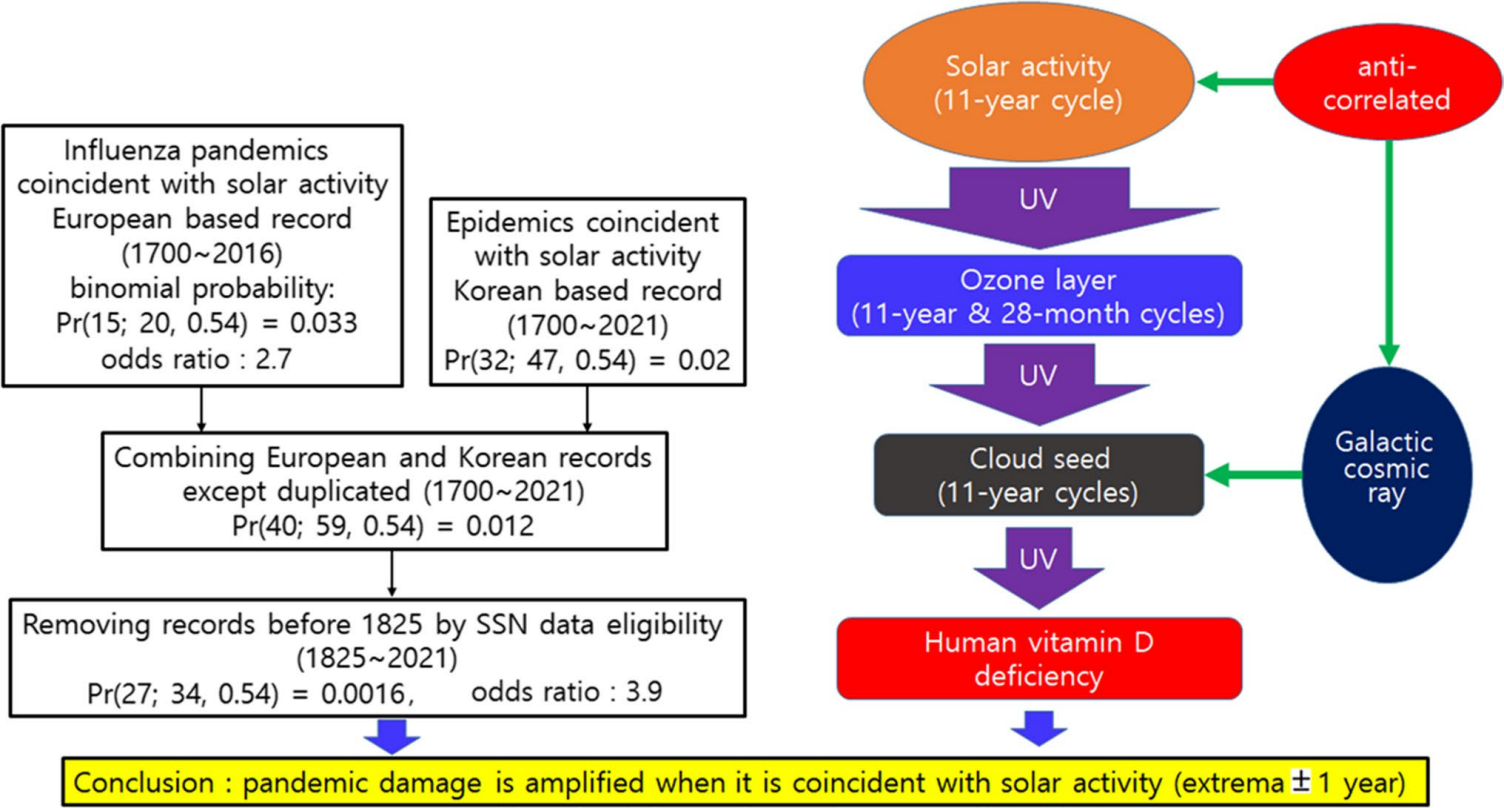
Flow diagram of pandemic record relation with solar activity and schematics of the physical mechanism that explains the causality

It is not conclusive to conclude that there is no correlation between the extrema of the sunspot cycle and the occurrence of pandemics based only on data from Europe. The correlation increases when a recent epidemic outbreak and non-European epidemic records are added. In particular, looking at data from 1825 onwards, the possibility that these two phenomena may be related should be included. Humans are not the only species influenced by solar cycles; the Norwegian insect population varies according to the solar cycle, which can be related to UV exposure (Vidar et al. 2004). Ultraviolet rays from the sun are blocked by the ozone layer in the stratosphere and cloud seeds in the low atmosphere. According to the solar cycle, ozone thickens at the maximum point of the sunspot number and reduces the number of incident UV rays. At the minimum point of the sunspot number, cloud seeds become denser and the incident amount of ultraviolet rays decreases because cloud seed production is accelerated by galactic cosmic rays. Since vitamin D is involved in the immune response to infectious diseases, vitamin D deficiency, which becomes more severe when the incident amount of ultraviolet rays decreases, can play a role in amplifying the damage caused by infectious diseases. There are two ways to consider the relationship between vitamin D and pandemic occurrence, leading to this conclusion, as summarized in Fig. 4. The first is the coincidence of pandemic outbreaks with sunspot number extrema, and the second is the underlying physical mechanism of UV generation and attenuation through the sun, ozone, and cloud seeds. The unique feature of this study comparing previous studies is summarized in Table 2, which shows that only this study includes the effect of cloud seeds as UV attenuation medium changing according to the solar cycle so that the pandemic occurrence at sunspot number minimum can be explained.

**Table 2:** Comparison of solar cycle–pandemic studies

Study (reference)	Covering years	Mechanism	Max or min of solar cycle	Summary
Hope-Simpson 1978	1919–1971	Not mentioned	Max only	The first recognition of influenza
Hoyle and Wickramasinghe 1990	1761–1990	Electric field of solar wind moves virus	Max only	Extended time and first suggested mechanism
Cannell et al. 2008	not available	Vitamin D	Max only	First suggestion of vitamin D as mechanism
Hayes 2010	1700–2000	Vitamin D, ozone	Max only	Vitamin D and ozone as a mechanism but the cloud seed effect is missing
Qu 2016	1700–2009	Vitamin D, mutation, birds movement, etc	Max and min ± 1	Both max and min are considered, but none of the suggested mechanisms is completed
This study	1700–2020	Vitamin D, ozone, cloud seed	Max and min ± 1	First to explain for both max and min, verification of the cloud seed effect is needed

The outbreak of severe acute respiratory syndrome (SARS) in 2003 is an example in which a severe infectious disease occurred at a non-extremum of the sunspot number, the damage of which was less severe than that of a milder virus outbreak at an extreme number of sunspots, such as the H1N1 flu pandemic (2009). One can imagine that the damage would be devastating if a virus as fatal as SARS occurred at the sunspot number extremum. Indeed, COVID-19 was fatal as SARS occurred at the sunspot number minimum, and the damage was devastating. The most significant limitation of this study is that the evidence of the cloud seeds variation and its effect on UV attenuation is lacking. Therefore, further extensive data analysis on this aspect is required. On the other hand, the ozone layer data analyzed in this study showed a 28-month periodicity by the QBO. Since the incident amount of ultraviolet rays is lowest at the point where the two kinds of extrema from the 11-year period of the solar cycle and the quasi-2-year (28-month) period of the stratosphere meet, this can be related to the cause of past epidemics occurring within one year before or after the sunspot extrema. As shown in Fig. 3. The 2-year periodicity tends to be as large as the 11-year periodicity; thus, it can be one of the reasons that most past pandemics ended within two years. This study leads to three important perspectives.

- Vaccination is found as a necessary but not a sufficient means to cope with COVID-19 (Coccia 2022b). Prevention of vitamin D deficiency through daily intake of vitamin D supplements is an effective countermeasure against infectious diseases, including COVID-19. Evidence showing taking dietary vitamin D is effective against the pandemic is the survival case of Norwegian Sea Sami, whose main food was vitamin D-rich oily fish, upon the deadly spread of bubonic plague in 1349, where 70% Norwegian have perished.
- Scientific community and health organizations need to establish a generic monitoring system of ozone and cloud seed as well as the global blood level of vitamin D for the understanding and prevention of the periodic pandemic occurrences.
- The COVID-19 outbreak can last up to 2 years, but it is highly likely to weaken in the third year. However, there was a case of abnormal stratospheric QBO suspected of being affected by climate change; therefore, the certainty of the future forecast is limited (Newman et al. 2016).

## Data Availability

Not available.

## Materials availability

Not available.
